# Peer Problems and Low Self-esteem Mediate the Suspicious and Non-suspicious Schizotypy–Reactive Aggression Relationship in Children and Adolescents

**DOI:** 10.1007/s10964-019-01125-9

**Published:** 2019-09-13

**Authors:** Keri Ka-Yee Wong, Adrian Raine

**Affiliations:** 1 Department of Psychology and Human Development, University of College London, London, WC1CH 0AA UK; 2 Departments of Criminology, Psychiatry, and Psychology, University of Pennsylvania, Philadelphia, 19104 PA USA

**Keywords:** Schizotypy, Reactive aggression, Proactive aggression, Suspiciousness, Paranoia, Childhood

## Abstract

The relationship between schizophrenia and violence has been well-established. Yet very little prior research exists on the factors that might explain the nature of this relationship and even fewer studies seek to clarify the etiology of aggressive behavior in adolescents with specific features of schizotypal personality that might help improve the specificity of intervention. The current study tested whether one dimension of schizotypy alone (i.e., the ‘suspicious’ feature) or the other 8 dimensions (i.e., the ‘non-suspicious’ features) were particularly associated with aggressive behaviors (reactive and proactive aggression), and if peer problems and low self-esteem mediated these relationships. A serial multiple mediation model testing the hypothesized flow from suspicious and non-suspicious schizotypy to peer problems to low self-esteem and to increased aggression was tested in Hong Kong schoolchildren aged 8- to 14-years (*N* = 1412; *M*_age_ = 11.47, *SD* = 1.67 years, female = 47.6%). Increased suspicious and non-suspicious schizotypal features were found to be independently associated with increased reactive aggression, but not proactive aggression. Children with high levels of suspicious schizotypy and non-suspicious schizotypy were more likely to have poor peer problems and low self-esteem concurrently, which in turn was associated with reactive aggression only. This explanatory model suggests that future longitudinal intervention studies that enhance self-esteem in schizotypal adolescents may potentially reduce co-morbid reactive aggressive behaviors too.

## Introduction

Schizophrenia is a disabling multidimensional disorder that is thought to be a risk factor for violence and criminal behavior. Increased rates of violence in schizophrenia have been documented in patients with schizophrenia who are on average 7 times more likely to commit serious violence like homicide than controls (Eronen et al. 1996; Fazel et al. 2009) and 20%–33% more likely to be victims of violent crime compared to the general population (de Vries et al. 2018). Conversely, exhaustive reviews have documented considerable support for raised rates of serious violence in incarcerated populations (Keers et al. 2014), research on which until recently has been hindered by ethical concerns over stigma (Torrey 2011). While drug and alcohol use in part explains this relationship, an attenuated link remains even after controlling for substance abuse (Fazel et al. 2009), suggesting that processes other than substance use may explain why schizophrenia is associated with violence. Thus, although the schizophrenia and violent crime relationship has been well-established over the last few decades (Raine et al. 2006), to prevent and understand the etiology of this disabling condition requires a more fine-grained examination of the causal factors of this relationship developmentally.

Understanding the development of schizophrenia is in part predicated on understanding the development of attenuated forms of this disorder in childhood and adolescence (Linscott and Van Os 2013). Evidence from clinical (Kendler and Diehl 1993) and community (Raine et al. 1994) research using schizotypal personality disorder as a theoretical framework has informed the etiology of schizophrenia-spectrum disorders, yet there is a dearth of research on attenuated forms of schizophrenia in childhood and adolescence (Debbané et al. 2013). Arguably, developmental research in adolescence with schizotypal personality, which shares phenotypic features with schizophrenia before the onset of schizophrenia-spectrum disorders, could lay the foundation for a better understanding of adult schizophrenia (Wong and Raine 2018). Thus, to fully understand the schizophrenia-violence relationship, one should ask the question: does an attenuated form of this link, schizotypy and aggression, exist earlier in development?

For adults, increased antisocial personality has been documented in studies of schizotypal patients (Freedman et al. 2007). In children and adolescents, findings are particularly sparse. Increased violence has been documented in both paranoid and schizotypal adolescents (Johnson et al. 2000), while psychotic symptoms at age 11 have been associated with adult violence at age 26 in those with schizophreniform disorder (Arseneault et al. 2003). In a recent prospective longitudinal study, children left home alone at age 3 have also been found to report higher levels of psychotic-like symptoms and schizotypal traits at age 17 and self-reported crime and schizotypal traits at age 23 compared to children reared by parents and siblings/relatives (Wong et al. 2018). However, prior studies have not examined this relationship in children younger than 11 years-old. To our knowledge, only two prior studies have examined this relationship, with both observing an aggression-schizotypy relationship in community-residing children (Raine et al. 2011; Seah and Ang 2008). However, no study has examined this relationship in a more nuanced manner to establish whether specific symptoms, such as paranoid suspicious symptoms (which is one dimension in schizotypy and the most common symptom in patients with schizophrenia) are especially related to violence, which would help inform early intervention strategies. Taken together, initial studies on children and adolescents suggest that a meaningful link between schizotypy and aggression does exist, but which subtype of schizotypy and aggression are most related?

One on-going impediment to research on childhood schizotypy and its individual subtypes like paranoia has been the lack of child-appropriate dimensional assessments for young children. Two new psychometrically robust self-report instruments appropriate for children aged 8 years and above have recently been developed to help address this barrier: The Schizotypal Personality Questionnaire–Child (SPQ-C), a downward extension of the adult 3-factor SPQ (cognitive-perceptual, interpersonal, and disorganized deficits) modeled on the nine DSM features of schizotypal personality disorder (Raine et al. 2011), and the Social Mistrust Scale (SMS), the first detailed dimensional assessment tool specifically for childhood suspicions only (Wong et al. 2014). Children are asked to endorse (no/sometimes/yes) items like: “others try to harm me at school/home” and “have you ever thought that other people are following you or spying on you at school/home?”. These instruments together offer a new window into exploring the correlates of, and the etiological theoretical questions in, the field of schizophrenia-spectrum disorders.

A further unresolved issue concerns whether schizophrenia-spectrum disorders are associated with all forms of aggression or specific to a subtype of aggression, an important distinction that could increase the specificity of interventions. Studies on adults have yet to address this issue. Conversely, three studies on children have addressed this question, suggesting that schizotypy is associated more with reactive retaliatory aggression than proactive instrumental aggression (Raine et al. 2006; Raine et al. 2011; Seah and Ang 2008). Conceivably, the type of aggression exhibited by schizotypal children may be less linked to the planned, regulated, proactive forms of aggression frequently associated with psychopathy (Raine et al. 2006; Trotman et al. 2006). Instead, aggression manifested by schizotypal children may be more impulsive and emotional in nature, consisting of unplanned reactively aggressive responses to provocation, which has implications on future assessments and interventions. Specifically, a recent meta-analysis of community adults (*N=* 23,444) has shown that paranoia/suspiciousness, which is one of the nine symptoms in schizotypy, is linked with more violence independent of other psychotic-like experiences and comorbid conditions like substance dependence (Coid et al. 2016). Initial cross-sectional evidence has also demonstrated that excessively suspicious children are more reactively aggressive, not proactively aggressive, compared with non-suspicious children (Wong et al. 2014) and childhood suspicions are heritable (Zhou et al. 2018). Though more extensive longitudinal studies are needed to assess the transmission of suspiciousness from parent to child pre- and post-natally (Wong and Esposito, 2019), there is also initial cross-sectional evidence for a social explanation model where persistently suspicious children are more likely to be victimized by peers and attribute hostile intent to others (Wong, 2015). Yet, why might childhood suspicions be associated with reactive aggression has yet to be investigated and may prove important for the development of early preventive interventions.

What may explain the schizotypy-aggression link in children and adolescents is unknown. In a prospective cohort study of UK adolescents, peer problems at age 8 and 10 years have been associated with psychotic-like symptoms at 12 years-old (Schreier et al. 2009). The only mediation study to date has shown that peer problems mediate the schizotypy-reactive aggression in a sample of socioeconomically diverse and ethnically Chinese Hong Kong children (Raine et al. 2011), though this finding has yet to extend to other socioeconomic/ethnic groups. It was hypothesized that schizotypal features elicit victimization from other children, which in turn predisposes to reactive aggression. It is plausible therefore that other social factors may mediate the schizotypy-aggression relationship, such as self-esteem, which can result from negative peer interactions including victimization and has been found to be related to both schizotypy and aggression (Fisher et al. 2013), however peer problems and self-esteem have not been examined together so the causal direction is unclear. A review of the findings on whether low self-esteem rather than high self-esteem leads to violence has been mixed (Ostrowsky 2010). In a cross-sectional study of 12-year-olds which accounted for discrepancies in self- and peer-ratings of self-esteem and social acceptance, low self-esteem and peer-rejection was more strongly associated with aggression in boys and not girls (Diamantopoulou et al. 2008). In another longitudinal study, self-esteem was unrelated to violent behaviors in early adolescent boys/girls and late adolescent boys, yet high self-esteem predicted violent behaviors in late adolescent girls (Ostrowsky 2009). In a meta-analytic study of 52 studies Chinese students (N = 82,358), low self-esteem was associated with aggression (i.e., physical aggression, anger, hostility, implicit and explicit aggression, but not verbal aggression), a relationship that was found to be stronger in adolescence (*r* = −0.24) than in young adulthood (*r* = −0.19) with no gender moderation (Teng et al. 2015). Thus, self-esteem in relation to gender and age differences deserve further clarification. Moreover, peer problems could also result in increased aggression via low self-esteem, a documented risk factor for both aggression and schizotypy (Donnellan et al. 2005; Ripoll et al. 2013) suggesting an alternative causal pathway is possible. Similarly, gender is another established moderating factor associated with both schizotypy and aggression, where males are more aggressive and schizotypal than females (Brennan and Alden 2006). Thus, in theory, repeated negative peer interactions may harm children’s self-esteem, resulting in reactive, retaliatory aggression in subsequent encounters with others, specifically to males. However, an alternative model flowing from existing low self-esteem resulting in poor peer problems should also be tested.

## Current Study

The aim of the current study is to advance the understanding of why children with schizotypal traits are more aggressive (reactive rather than proactive aggressive) and whether peer problems and low self-esteem could explain this relationship. Identifying potential mediators of the schizotypy-aggression relationship could pave way for future longitudinal intervention studies to reduce aggressive behaviors in adolescents with schizotypy. Five hypotheses were tested: First, does a downward extension of the schizophrenia-crime relationship exists and if so, it is hypothesized that community schoolchildren and adolescents with schizotypal features will be more likely to be aggressive (Hypothesis 1). Second, given the child and adolescent literature, this relationship will be stronger for reactive aggression than for proactive aggression (Hypothesis 2). Third, given that those with paranoid schizophrenia have a higher risk of violence than those with non-paranoid forms of schizophrenia (Brennan and Alden 2006), paranoid suspicious features of schizotypy will be more strongly related to reactive aggression than non-suspicious schizotypy (Hypothesis 3). Fourth, and central to the current study, a serial mediation model is hypothesized to flow from non-suspicious schizotypy and suspiciousness to peer problems to low self-esteem and in turn to aggression (Hypothesis 4), given the theoretical assumption that peer problems can lead to low self-esteem. Fifth, given the mixed gender and age findings with few studies examining other ethnic groups, gender, age and ethnicity were included as covariates in the serial mediation models of non-suspicious schizotypy and suspiciousness and aggression relationship to test for generalizability of the findings (Hypothesis 5).

## Methods

### Participants

1412 8- to 14-year-olds (*M* = 11.47 years, *SD=* 1.67, female = 47.6%) completed a battery of self-report questionnaires during a 50-minute whole-class testing session. This excluded children who opted out of the study (*n=* 31), those with learning disabilities (*n=* 1), and those without complete data on all study variables (*n=* 79). Mediation modeling were conducted on subsamples with data available on all variables. All international schools with a GCSE/IB curriculum in Hong Kong were invited to participate in the study as this matched with the equivalent UK schools recruited as part of a larger study (Wong et al. 2014). Participants were recruited from 8 primary and secondary international schools spanning the three major catchment areas in Hong Kong (e.g., Hong Kong Island, Kowloon, and New Territories). English was the primary language of teaching in classrooms. English was the most commonly spoken language at home (60.88%), but a large proportion of children were also bilingual and spoke Cantonese (19.02%) or Mandarin Chinese (10.98%) at home. The majority of participants were ethnically Chinese (51.39%), with British/Irish (12.95%), Asian British (8.54%), Mixed (8.82%) and Other ethnicities including French and Italian, (18.3%) were represented. The sample was predominantly from high affluent (81%) and medium affluent (18.6%) families in terms of the WHO definition of medium to high, as defined by the family affluence scale (Boyce et al. 2006). Just over half of the participants had at least one sibling (51.7%), was an only child (15.08%) and the remaining chose not to disclose this information. The majority of participants were from two-parent homes (86.75%), followed by single-parent homes (6.23%) and some with neither parent (0.14%). For some participants, they lived with other adults in the home such as a grandparent (14.09%) or a live-in nanny (58.38%), suggesting perhaps interesting familial socialization that may be different to most Western societies. To maximize participation, the method of informed passive consent in which schools acted in *loco parentis* but parents were given opportunities to decline their child’s participation before, during, and after the study was adopted. The study received ethical approval from The Cambridge Psychology Research Ethics Committee at the University of Cambridge (2011.42). A more detailed description of participants’ demographics can be found in Table 1.

**Table 1:** Participant demographics (*N* = 1412)

	%
Family structure	
Two parent	86.75
Single parent	6.23
Neither	0.14
Non-parental adults	
Grandparent	14.09
Nanny	58.38
Siblings	
At least one	51.70
None	15.08
Socioeconomic Status (FAS)	
Low	0.44
Medium	18.56
High	81.00
Ethnicity	
Chinese	51.39
British & Irish	12.95
Asian British Indian^a^	8.54
Korean	2.56
Japanese	1.64
Mixed^b^	8.82
Other^c^	12.95
Language spoken at home^d^	
English	60.88
Mandarin Chinese	10.98
Cantonese	19.02
Hindi	1.36
Japanese	0.93
Korean	2.23
German	0.22
French	0.50
Italian	0.29
Pakistani/Bengali/Punjabi/Gujarati	0.64

^a^Asian British Indian, Pakistani, Bangladeshi, Black or Black British African

^b^Mixed White & Black Caribbean, Black African, and Asian

^c^Australian, Polish, American, Canadian

^d^97.5% of the most common first languages spoken at home. Remaining % unreported have missing information

## Measures

### Non-suspicious Schizotypy

The Schizotypal Personality Questionnaire–Child (SPQC) is a 22-item yes/no child-appropriate self-report measure of schizotypal personality (Liu et al. 2019; Raine et al. 2011). To provide a measure of non-suspicious schizotypy, the author of the original SPQC agreed to exclude the four items assessing suspiciousness (i.e., 7, 9, 14, 17) and summed the remaining 18 items to measure non-suspicious schizotypy so as to measure the distinct contributions of non-suspicious schizotypy features with aggression. The remaining scale still consists of the original three factors: Cognitive-Perceptual (F1: “I sometimes feel distracted by far-off sounds that I’m not normally aware of”), Interpersonal (F2: “I tend to keep my feelings to myself”) and Disorganized (F3: “I sometimes act oddly”) features and had good internal reliability (*α* = 0.81). Data were available on 1327 individuals for the non-suspiciousness total score and subscales scores (F1 = 1422; F2 = 1388; F3 = 1417).

### Suspiciousness

This was assessed dimensionally using the Social Mistrust Scale (SMS) (Wong et al. 2014), a 12-item child-report measure of paranoid suspicions ranging from 0 to 24, where a higher score denotes higher levels of suspiciousness. Sample items include: “I worry too much about others trying to get at me at school” and “have you ever thought that people are following you or spying on you at home?” The SMS has a reading age appropriate for 8-year-olds and above and has good test-retest reliability (ICC = 0.80, *r* = 0.80), internal reliability (*α* = 0.75) and construct validity in this study. The SMS correlates with the SPQ-C four paranoid items at *r* = 0.49 and the non-suspicious SPQ-C 18-items at *r* = 0.53. Data available on the 1451 individuals.

### Reactive and Proactive Aggression

The Reactive-Proactive Questionnaire (RPQ) is a well-established measure of reactive provoked aggression (11 items) and proactive instrumental aggression (12 items) (Raine et al. 2006). The 23-item self-report was measured on a 3-point ordinal scale (Never (0), Sometimes (1), and Often (2)) resulting in a score ranging from 0 to 46. Sample items include: “How often have you gotten angry when others threatened you?” (reactive) and “How often have you had fights with others to show who was on top?” (proactive). Though both reactive and proactive aggression are highly correlated in this study (*r* = 0.61), regression analyses controlling for reactive and proactive aggression and vice versa were conducted to examine the specific relationships with suspicious and non-suspicious schizotypy. Reactive (*α=* 0.84) and proactive (*α* = 0.80) subscales showed good internal reliabilities in this study. Data were available on 1192 and 1194 individuals for the reactive and proactive subscales respectively.

### Peer Problems

Five items (two reverse-coded) from the original 25 item Strengths and Difficulties Questionnaire (SDQ) were summed to create the peer-problems subscale with modest internal reliability for this study (*α* = 0.56) (Goodman 1997). A sample item is: “I am usually on my own. I generally play alone or keep to myself.” Previous studies have also found moderate internal reliability (*α* = 0.52) and test-retest reliability (*r* = 0.45) (Yao et al. 2009). Data were available on 1441 individuals.

### Self-esteem

The Rosenberg Self-Esteem Scale (RSES) is a 10-item self-reported measure of self-esteem with good internal reliabilities (*α* = 0.77 to 0.88) and test-retest reliabilities (*r* = 0.82 to 0.88) (Rosenberg 1965). A sample item is: “At times I think I am no good at all”. Internal reliability in this study was good (*α* = 0.81). Data were available on 1299 individuals.

### Demographics

Children self-reported their date of birth (N = 1487), gender (N = 1534), ethnicity (N = 1519), and socioeconomic status as measured by the 4-item Family Affluence Scale (FAS), which ranges from 0 to 9 (0–3 = low, 4–6 = medium, 7–9 = high affluence) (Boyce et al. 2006). Sample items include: “Does your family own a car, van, or truck?” (No [0], Yes One [1], Yes two or more [2]) or “During the last 12 months, how often did you travel away on holiday with your family” (Not at all [0], Once [1], Twice [2], More than twice [3]). Due to the small sample size in each of the other non-Chinese ethnic groups, ethnicity was dichotomized into Chinese (0) vs non-Chinese (1). Data were available on 1434 individuals.

### Data Analysis

#### Missingness

The full analytic sample size and proportion missing for each variable can be found in Table 2. A missing values analysis was conducted on all key study variables to determine the extent of missingness. Little’s MCAR test (Little 1988) was non-significant, χ^2^ (343) = 291.82, *p* = 0.979, suggesting that the assumption of missing completely at random is not violated. Thus, complete data on each variable were used in all analyses.

**Table 2:** Descriptive statistics for all study variables

	1	2	3	4	5	6	7	8	9	10	11	12	13
1. Age	–												
2. Gender (male [0], female [1])	0.02	–											
3. Ethnicity (Chinese [0] vs. Non-Chinese [1])	−0.11^**^	0.08^**^	–										
4. Socioeconomic status (low [0], medium/high affluence [1])	−0.02	0.01	0.09^**^	–									
5. Suspiciousness (SMS)	−0.09^**^	−0.04	0.03	−0.03	–								
6. Non-suspicious Schizotypy (SPQC-18)	−0.**04**	0.09^*^	0.03	−0.03	0.53^**^	–							
7. Cognitive-perceptual (F1)	−0.08^**^	0.03	0.04	0.03	0.42^**^	0.76^**^	–						
8. Interpersonal (F2)	−0.07^**^	0.02	−0.05	−0.07^**^	0.40^**^	0.75^**^	0.35^**^	–					
9. Disorganized (F3)	−0.03	0.11^*^	0.06^**^	−0.03	0.42^**^	0.82^**^	0.45^**^	0.44^**^	–				
10. Reactive aggression (RPQ)	0.04	−0.07^**^	0.04	0.02	0.35^**^	0.39^**^	0.28^**^	0.28^**^	0.31^**^	–			
11. Proactive aggression (RPQ)	0.09^**^	−0.08^**^	−0.01	0.03	0.26^**^	0.27^**^	0.23^**^	0.18^**^	0.23^**^	0.61^**^	–		
12. Peer problems (SDQ)	−0.11^**^	−0.06^**^	−0.09^**^	−0.06^**^	0.46^**^	0.46^**^	0.27^**^	0.45^**^	0.38^**^	0.24^**^	0.13^**^	–	
13. Self-esteem (RSE)	−0.06^**^	−0.13^**^	0.05^*^	0.04	−0.38^**^	−0.42^**^	−0.27^**^	−0.36^**^	−0.37^**^	−0.22^**^	−0.14^**^	−0.40^**^	–
*α*	–	–	–	–	0.75	0.80	0.66	0.64	0.75	0.84	0.80	0.56	0.81
Mean (*SD*)	11.50 (1.71)	0.47 (0.50)	1.80 (0.41)	0.49 (0.50)	3.71 (3.43)	6.96 (4.14)	2.33 (1.77)	2.34 (1.67)	2.31 (1.84)	6.13 (4.09)	2.05 (2.76)	2.26 (1.79)	19.50 (4.51)
Range	8–14	0–1	0–2	0–1	0–17	0–18	0–6	0–6	0–6	0–22	0–22	0–10	3–30
Kurtosis	−0.54	–	–	–	1.10	−0.51	−0.94	−0.81	−1.01	.55	5.67	.90	.08
Analytic sample (N)	1487	1534	1519	1434	1451	1327	1422	1388	1417	1192	1194	1441	1299
Missing (%)	3.13	0.07	1.04	6.58	5.47	13.55	7.36	9.58	7.69	22.35	22.22	6.12	15.38

Spearman’s rho and Kendall’s Tau-b correlations where appropriate

^*^p < 0.05; ^**^p < 0.01

#### Correlations and regressions

The relationships between study variables were first examined using Spearman’s rho correlations (continuous/skewed) and Kendall’s tau-b (continuous/categorical) in (SPSS 25.0 2017). Next, hierarchical multiple regressions with non-suspicious schizotypy and suspiciousness as outcome variables, and proactive and reactive aggression as independent variables were entered in two steps to assess whether non-suspicious schizotypy and suspiciousness were independently more strongly related to reactive than to proactive aggression. Finally, above and beyond the initial link between the suspiciousness and reactive aggression relationship and the non-suspicious schizotypy and reactive aggression relationship, the extent to which both were mediated by peer problems and self-esteem were examined as percentages by entering the non-suspicious schizotypy and suspiciousness independently in step 1 and mediators in step 2 using a hierarchical multiple regression.

#### Mediation analyses

Hierarchical multiple regressions were conducted to assess the non-suspicious schizotypy and suspiciousness with aggression link controlling for peer problems and self-esteem to both establish them as potential mediators and to assess the extent of mediation. Standardized beta coefficients (*β*), adjusted R-squared (*R*^*2*^_adj_) and R-squared change (*ΔR*^*2*^) statistics were reported.

#### Serial multiple mediation models

The PROCESS v.3 SPSS macro was used to test the theoretical causal pathways from the three factors of non-suspicious schizotypy (x_1_) to reactive aggression (y), first through peer problems (m_1_) and then to self-esteem (m_2_) (Hayes 2017). While full mediation is unlikely, partial mediation is established when indirect pathways through the mediator are significant (i.e., the confidence interval excludes 0) and the direct pathway between the independent variable (X) and dependent variable (Y) remains significant. Alternative model analyses were conducted to test the same models for non-suspiciousness (x_1_) and suspiciousness (x_2_) as separate independent variables flowing through the mediators peer problems and self-esteem (m1, m2) and vice versa (m2, m1) to predict reactive aggression. Finally, models were rerun by including background demographic variables as covariates to examine whether the serial mediation effects still hold after this control. To test the significance of indirect effects, bias-corrected confidence intervals (CIs) for indirect effects were generated by taking 10,000-bootstrap samples as recommended by Hayes (2017). Whereas normal theory assumes that the sampling distribution of indirect effects are normally distributed, bootstrapping was used because it constitutes a non-parametric resampling procedure that makes more realistic assumptions on the sampling distribution and estimations. Estimations are based on the raw data and account for non-normally distributed variables (Hayes 2017).

## Results

### Covariates

Males reported higher levels of reactive (*r* = −0.07, *p* = 0.003) and proactive aggression (*r* = −0.08, *p* = 0.003), peer problems, and lower self-esteem compared with females. Females were overall more schizotypal than males as reflected on the total non-suspicious schizotypy score (*r* = 0.08, *p* < 0.001), but specifically, this relationship was driven by the disorganized factor 3 (*r* = 0.11, *p* < 0.001) (see Table 2). Age as a categorical variable was only associated with proactive aggression (*r* = 0.09, *p* < 0.001) but not with reactive aggression (*r* = 0.04, *p* = 0.071). Ethnicity was unrelated to both reactive (*p* = 0.134) and proactive aggression (*p* = .779) but non-Chinese participants reported more non-suspicious schizotypy features, particularly disorganized, than their Chinese counterparts (*r* = 0.06, *p* = 0.009). Socioeconomic status is not associated with suspiciousness and aggression (*ps* > 0.05) but associated with interpersonal deficits of non-suspicious schizotypy (*r* = −0.07, *p* = 0.003). Consequently, gender was included as covariate of the suspicious/non-suspicious schizotypy and reactive aggression relationship and age was included for the proactive aggression serial mediation analyses.

### Non-suspicious Schizotypy, Suspiciousness, and Aggression Correlations

Correlations between study variables are shown in Table 2. Non-suspicious schizotypy was more strongly associated with reactive (*r* = 0.39, *p* < 0.001) than with proactive aggression (*r* = 0.27, *p* < 0.001) (Steiger statistic, *z* = −4.88, *p* < 0.001). Similarly, suspiciousness was more strongly associated with reactive (*r* = 0.35, *p* < 0.001) than proactive aggression (*r* = 0.26, *p* < 0.001) (Steiger statistic, *z* = −3.2, *p* = 0.001). Interestingly, the similarities in the magnitude of correlations between non-suspicious schizotypy and suspiciousness with both reactive and proactive aggression suggests that both paths are significant and that paranoid traits alone are equally associated with elevated aggression as the remaining eight non-suspicious schizotypy symptoms.

### Non-suspicious Schizotypy and Aggression Subtypes

To examine the relative association between non-suspicious schizotypy and each of the aggression subtypes, hierarchical multiple regressions with proactive aggression (step 1) and reactive aggression (step 2) were entered as independent variables and non-suspicious schizotypy as a dependent variable. Non-suspicious schizotypy was still significantly associated with reactive aggression (*β* = 0.41, *p* < 0.001), controlling for proactive aggression (*R*^*2*^_*adj*_ = 0.15, *F*[2, 1318] = 117.298, *p* < 0.001). The same results were found for the relationship between reactive aggression and each of the non-suspicious schizotypy factors: cognitive-perceptual (*β* = 0.37, *F*[2, 1318] = 111.143, *p* < 0.001, *R*^*2*^_adj_ = 0.14), interpersonal (*β* = 0.11, *F*[2, 1318] = 80.84, *p* < 0.001, *R*^*2*^_adj_ = 0.11), and disorganized (*β* = 0.41, *F*[2, 1318] = 114.24, *p* < 0.001, *R*^*2*^_adj_ = 0.15).

Conversely, controlling for reactive aggression (step 1) rendered the non-suspicious schizotypy and proactive aggression relationship non-significant (*β*: SPQ total = −0.02, *p* = 0.69; cognitive-perceptual = 0.01, *p* = 0.83; interpersonal = −0.07, *p* = 0.25; disorganized = −0.03, *p* = 0.67), indicating the primacy of reactive aggression (and not proactive aggression) in relation to non-suspicious schizotypy.

### Suspiciousness and Aggression Subtypes

Suspiciousness was entered as the dependent variable predicted by proactive aggression (step 1) and reactive aggression (step 2). The suspiciousness and reactive aggression relationship remained significant (*β* = 0.26, *p* < 0.001) controlling for proactive aggression (*R*^*2*^_adj_ = 0.11, *F*[2, 1276] = 81.20, *p* < 0.001). Similarly, controlling for reactive aggression (step 1) rendered the suspiciousness and proactive aggression relationship non-significant (*β* = 0.08, *p* = 0.17, Δ*R*^*2*^ = 0.001), again indicating that suspiciousness features were predominantly related to reactive and not proactive aggression.

### Serial Multiple Mediations

#### Non-suspicious schizotypy and reactive aggression

**Fig. 1:**
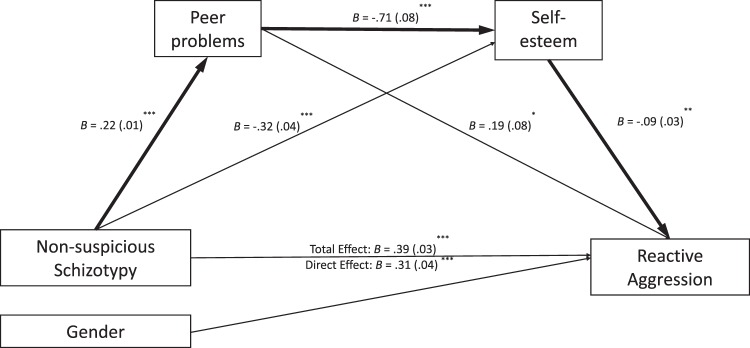
A serial mediation model flowing from non-suspicious schizotypy (SPQC18) to peer problems, self-esteem and reactive aggression controlling for gender in every path (N = 962). ^***^p < 0.001, ^**^p < 0.01, ^*^p < 0.05. *B* = unstandardized coefficients, SE standard errors in brackets. *R*^*2*^ = 0.18, *F*(4, 957) = 51.71, *p* < 0.001

Figure 1 presents the hypothesized serial mediation model of the non-suspicious schizotypy and reactive aggression relationship flowing through peer problems (indirect effect: *B* = 0.05, 95% CI [0.011, 0.089], *p* < 0.05) and self-esteem (indirect effect: *B* = 0.02, 95% CI [0.002, 0.048], *p* < 0.05), demonstrating a partial mediation (indirect effect: *B* = 0.01, 95% CI [0.001, 0.020], *p* < 0.05) as the direct effect remained significant (*B* = 0.30, *SE* = 0.04, *t*(960) = 8.52, *p* < 0.001). Significant contrasts were found between indirect effects through both peer problems and self-esteem and the indirect effects through self-esteem alone (*B* = −0.02, *p* < 0.05), suggesting that schizotypal tendencies partially predispose to peer problems, which result in lower self-esteem and increased reactive aggression, although the effects were larger through self-esteem alone. The serial mediation effects remained significant after including gender as a covariate (indirect effect: *B* = 0.01, 95% CI [0.003, 0.024], *p* < 0.05).

#### Non-suspicious schizotypy and proactive aggression

A serial multiple mediation analysis was repeated for the non-suspicious schizotypy and proactive aggression relationship flowing through peer problems (indirect effect: *B* = 0.02, *ns*) and self-esteem (indirect effect: *B* = 0.02, *ns*). The overall serial mediation pathway was non-significant (indirect effect: *B* = 0.01, *ns*).

#### Suspiciousness and reactive aggression

**Fig. 2:**
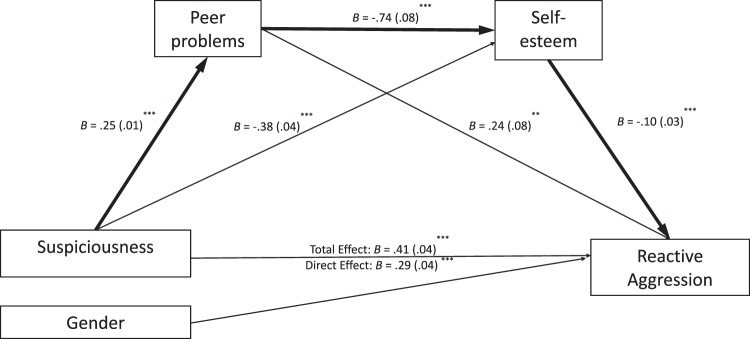
A serial mediation model flowing from suspicious schizotypy (SMS) to peer problems, self-esteem and reactive aggression controlling for gender in every path (N = 1039). Notes. ^***^p < 0.001, ^**^p < 0.01, ^*^p < 0.05. B unstandardized coefficients, SE standard errors in brackets. *R*^2^ = 0.15, F(4, 1034) = 45.23, *p* < 0.001

Figure 2 presents the hypothesized causal flow from suspiciousness to peer problems (indirect effect: *B* = 0.07, 95% CI [0.021, 0.114], *p* < 0.001) to self-esteem (indirect effect: *B* = 0.03, 95% CI [0.009, 0.061], *p* < 0.001) to reactive aggression. Results demonstrated partial mediation (indirect effect: *B* = 0.02, *SE* = 0.01, 95% CI [0.004, 0.028], *p* < 0.001) as the direct effect remained significant (direct effect: *B* = 0.30, 95% CI [0.217, 0.378], *p* < 0.001). Contrasts between indirect paths suggests that the mediation effect of self-esteem on the suspiciousness and reactive aggression relationship was stronger than the serial mediation through peer problems and self-esteem (*B* = 0.02, *p* < 0.05). Suspiciousness, therefore in part predisposes to poor peer relations, which reduces self-esteem and results in reactive aggression, with larger effects through self-esteem alone. The serial mediation effects remained significant even after including gender as a covariate (indirect effect: *B* = 0.02, 95% CI [0.007, 0.033], *p* < 0.05).

#### Suspiciousness and proactive aggression

A serial multiple mediation analysis was repeated with proactive aggression as the dependent variable and demonstrated that peer problems and self-esteem together marginally mediated the suspiciousness and proactive aggression relationship (indirect effect: *B* = 0.01, *SE* = 0.00, 95% CI [0.0004, 0.0167], *p* < 0.001), however the indirect effect through peer problems was non-significant (indirect effect: *B* = 0.02, *ns*).

### Alternative Serial Mediation Model Analyses

**Fig. 3:**
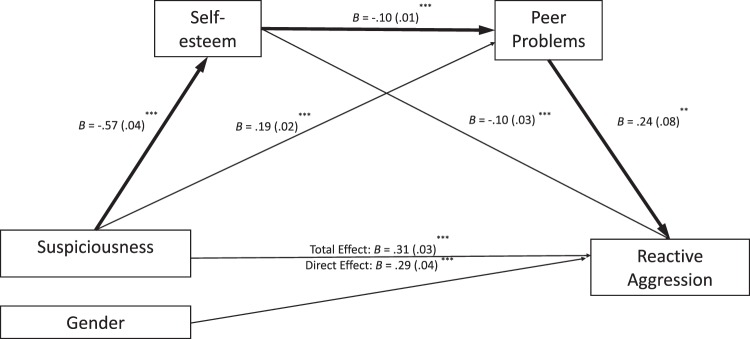
An alternative serial multiple mediation model from suspiciousness to self-esteem, then to peer problems, and reactive aggression controlling for gender at every path (N = 1039). Notes. ^***^p < 0.001, ^**^p < 0.01, ^*^p < 0.05. B unstandardized coefficients, SE standard errors in brackets. *R*^2^ = 0.15, F(4, 1034) = 45.23, *p* < 0.001

**Fig. 4:**
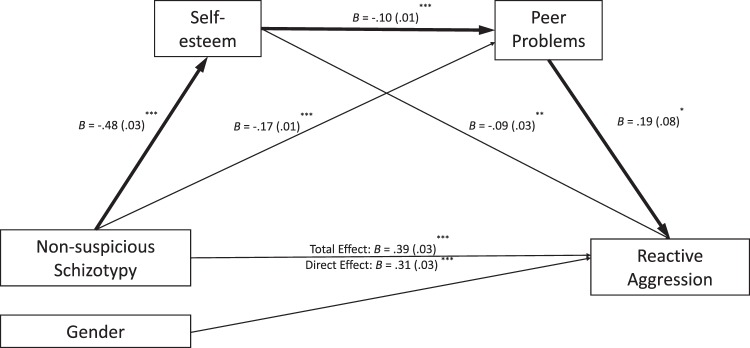
An alternative serial mediation model flowing from non-suspicious schizotypy (SPQC18) to self-esteem to peer problems, then to reactive aggression controlling for gender in every path (N = 962). ^***^p < 0.001, ^**^p < 0.01, ^*^p < 0.05. B unstandardized coefficients, SE standard errors in brackets. *R*^*2*^ = 0.18, *F*(4, 957) = 51.71, *p* < 0.001

Given previous studies described in the introduction on alternative pathways (from self-esteem to peer problems) and the cross-sectional nature of the study, alternative models with reversed mediators were tested to see whether this causal flow would produce the same results. The theoretical causal model consisted of a pathway flowing from non-suspicious schizotypy to low self-esteem, to peer problems, and in turn to reactive aggression. The same model was repeated for suspiciousness as the independent variable. Both serial mediation models were still significant (see Figs. 3 and 4). However, for both non-suspicious schizotypy and reactive aggression and suspiciousness and reactive aggression relationships, the flow through peer problems to low self-esteem was stronger than when the flow was from low self-esteem to peer problems (*B* = −0.71, *p* < 0.001 vs *B* = −0.10, *p* < 0.01). This suggests that self-esteem and peer problems partially drive the mediation, but that self-esteem drives the mediation more strongly in both non-suspicious schizotypy and suspiciousness and reactive aggression relationships.

### Extent of Serial Multiple Mediations

#### Non-suspicious schizotypy and reactive aggression

**Fig. 5:**
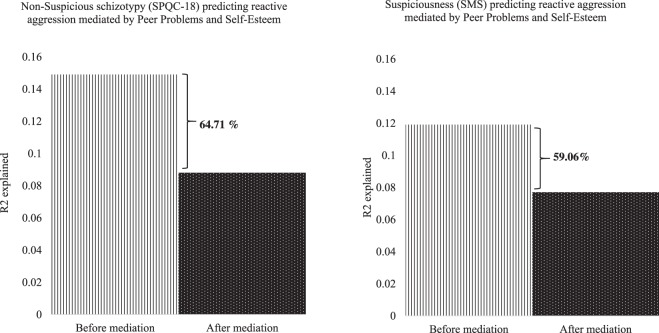
Bar graphs showing the variance explained (*R*^*2*^) by peer problems and self-esteem in the non-suspicious schizotypy and suspiciousness and reactive aggression relationship

A hierarchical multiple regression model with reactive aggression as the dependent variable controlling for peer problems and self-esteem (entered in step 1) and predicting non-suspicious schizotypy (in step 2) found that both peer problems (*β* = 0.10, *p* < 0.01) and self-esteem (*β* = −0.08, *p* < 0.05) were significant mediators (*R*^2^_adj_ = 0.17, *F*[1958] = 72.62, *p* < 0.001), explaining 59.06% of the non-suspicious schizotypy and reactive aggression relationship (see Fig. 5).

#### Suspiciousness and reactive aggression

Similarly, A hierarchical multiple regression model with reactive aggression as the dependent variable was predicted by peer problems and self-esteem (in step 1) and suspiciousness (in step 2) found that peer problems (*β* = 0.12, *p* < 0.001) and self-esteem (*β* = −0.10, *p* < 0.001) were significant mediators (*R* = 0.38, *R*^*2*^_adj_ = 0.13.9, *F*[3, 1035] = 56.76, *p* < 0.001), explaining 64.71% of the suspiciousness and reactive aggression relationship (see Fig. 5).

### Serial Mediation Models with Covariates

Gender and age were tested as potential covariates for all serial mediation model pathways between non-suspicious schizotypy and suspiciousness to aggression. All serial mediation pathways after including these covariates improved variance explained for the non-suspicious and reactive aggression relationship (no covariates: *R*^*2*^ = 0.17, *F*[3958] = 63.90, *p* < 0.001; with covariates: *R*^*2*^ = 0.18, *F*[5956] = 41.84, *p* < 0.001) and the suspiciousness and reactive aggression relationship (no covariates: *R*^*2*^ = 0.14, *F*[3,1035] = 56.76, *p* < 0.001; with covariates: *R*^*2*^ = 0.15, *F*[5,1033] = 37.37, *p* < 0.001). All serial mediation pathways remained significant controlling for covariates, suggesting that the relationships are not spurious.

## Discussion

Although past research has established a relationship between schizophrenia and violent crime, few studies have examined the causes of this relationship in its attenuated form–schizotypal traits and aggressive behaviors–and even fewer studies have taken a developmental approach to investigate younger community samples. A primary reason for this impediment is the stigma surrounding the negative connotation of being both dangerous and violent, whereby research into the attenuated schizotypy-aggression relationship may avoid. A second impediment has been the lack of developmental studies due to the scarcity of child-appropriate dimensional assessment tools into younger populations, a potentially important area of development that may inform early assessments and preventive interventions. The current study addressed these concerns by testing several serial mediation models to understand theoretically whether both peer problems and low self-esteem mediated the suspiciousness and non-suspicious schizotypy- aggression relationship in a large sample of ethnically diverse Hong Kong schoolchildren aged 8 to 14 years, using dimensional child-appropriate assessments.

Consistent with previous studies of the schizotypy-aggression relationship (Raine et al. 2006, 2011; Seah and Ang 2008), this study demonstrated that Hong Kong schoolchildren with higher levels of non-suspicious schizotypal traits and suspiciousness reported higher levels of aggressive behaviors, specifically reactive aggression and not proactive aggression, confirming study Hypotheses 1 and 2. This is not surprising as the current study sample is fairly similar in age to the previous studies cited, though the current sample offers evidence from a more culturally diverse multi-ethnic international school sample. This finding concurs with the notion that children and adolescents with high levels of schizotypal traits, particularly, odd behaviors, poor interpersonal relations, suspicions may be more likely to have poor relationships with their peers and become victims of bullying resulting in retaliatory responses (Fisher et al. 2013; Raine et al. 2011; Schreier et al. 2009). Interestingly, this finding is also consistent with the adult clinical literature whereby patients with schizophrenia are more likely to be victims of violent crimes (de Vries et al. 2018). Though further comparison across different ethnic groups beyond Chinese and non-Chinese were not possible due to small sample size, future studies should explore this question further by recruiting larger numbers in each ethnic group.

Contrastingly, Hypothesis 3 that suspicious schizotypy alone would be more associated with aggression than non-suspicious schizotypy, which was predicated on prior work (Brennan and Alden 2006; Dodge 2006; Tone and Davis 2012), was not supported. One explanation for this null finding may be that the SPQ-C measure of non-suspicious schizotypy reflects eight features of schizotypy as opposed to the single feature of suspiciousness in the SMS and encapsulates several different pathways from schizotypy to aggression. For example, the eccentricities of odd behavior and odd speech may make children stand out as targets for teasing (Kelleher et al. 2012). Blunted affect and lack of close friends may result in unpopularity, social exclusion, and vulnerability to bullying (Wong, 2015). Odd beliefs and magical thinking are schizotypal analogs of delusions, and delusions have been associated with violence (Coid et al. 2013). These different schizotypal features may together produce a relationship with aggression equally as strong as suspiciousness alone, thus nullifying the hypothesis that suspiciousness would be more strongly related to reactive aggression than non-suspicious schizotypy.

This study’s findings provide initial support for a putative causal model moving from schizotypy to peer problems to reduced self-esteem to reactive aggression, confirming Hypothesis 4. Odd, schizotypal children are more likely to be victimized than non-schizotypal children, resulting in low self-esteem, which in turn is a documented risk factor for aggression and delinquency (Diamantopoulou et al. 2008). The path from reactive aggression to self-esteem was not strong, albeit significant explaining 59.06% to 64.71% of the variance for the non-suspicious schizotypy and suspiciousness to reactive aggression models, respectively should be interpreted with caution. If replicated, results could have clinical implications for reducing aggression in schizotypal children. Reducing school bullying requires effortful intervention at a broad community level. Intervention to enhance self-esteem at an individual therapeutic level may equally mitigate reactive aggression in children presenting with schizotypal traits. For instance, multidisciplinary mentoring programs where students benefit from mentors have shown improvements in mentored students’ self-esteem, and in some cases, prevented childhood bullying and the exacerbation of depression (King et al. 2002).

Covariate analyses controlling for gender and age show that the multiple serial mediation models flowing from non-suspicious schizotypy and suspiciousness through peer problems and self-esteem to reactive aggression were still significant, which amplify the findings in a significant way. While substance abuse has been argued to partly account for the schizophrenia-violence relationship in previous research (Fazel et al. 2009), this seems an unlikely explanation for children and adolescents of this age group, although older children in this sample could be beginning to initiate alcohol and cannabis use. Nevertheless, the fact that age did not moderate findings indicates that findings apply to young children, a group unlikely to be abusing drugs, suggesting that the schizotypy–aggression relationship observed here is an unlikely confound of substance abuse. This study’s findings demonstrate that children with features of schizotypy are at heightened risk of reactive aggression and that peer problems and self-esteem are candidate theoretical causal mechanisms in explaining this relationship and deserve further replication with a longitudinal design.

Several study caveats need to be emphasized. First, while mediation analyses were conducted to test theoretical causal models in this cross-sectional study, randomized controlled trials that experimentally manipulate peer problems and self-esteem are required to establish causality. Second, mediation findings from East Asian children need to be generalized to Western populations, though this study begins to address differences in Chinese and non-Chinese ethnic groups, the sample of other ethnic groups was too small for meaningful comparison. Third, this sample with a maximal age of 14 may under-represent the strength of mediating effects in older teenagers, as this study shows that poor self-esteem and increased peer problems increase with age (see Table 2). Fourth, the reliance on a single informant self-report approach may inflate the relationships observed and future research drawing from teacher- and peer-ratings of behaviors may prove valuable (Diamantopoulou et al. 2008). Importantly, future research should test these relationships further with a longitudinal design.

In spite of these limitations, these initial findings help address the question of *why* schizotypy predisposes to aggression. By considering peer problems and low self-esteem as potential mediators that account for two-thirds of the suspiciousness/non-suspicious schizotypy and reactive aggression relationship, the current study’s findings provide meaningful pointers for modest interventions to reduce reactive aggression in children with schizotypal features. This study also adds to the literature by providing a theoretical framework appropriate for further investigation into the developmental risk and protective pathways in adolescence that could potentially inform the understanding of adult schizophrenia-spectrum disorders and criminal behaviors, an area of research that is paramount to the future development of early preventive interventions for society more broadly. Future research could benefit from recruiting a more ethnically- and socioeconomically-diverse sample of participants to further clarify the generalizability of these findings.

## Conclusion

Research on schizotypal personality traits has provided a valuable framework in which to understand the causes of disabling conditions like schizophrenia. While previous research has established the schizophrenia-crime relationship, few studies to date have investigated the schizotypy-aggression relationship, arguably its attenuated form, and the nature of this relationship as providing a potential theoretical framework through which to identify developmental risk/protective factors for early intervention. The current study addressed these gaps by conducting multiple serial mediation models to theoretically test whether peer problems and low self-esteem could explain why adolescents with schizotypal traits are more aggressive in a large sample of Hong Kong schoolchildren aged 8 to 14 years. Consistent with previous studies (Raine et al. 2006, 2011; Seah and Ang 2008), children with high levels of non-suspicious schizotypal traits and suspiciousness alone were also more reactively aggressive than proactively aggressive. The study findings advance the literature by demonstrating for the first time that non-suspicious schizotypy and suspiciousness alone are *both* independently and strongly associated with reactive aggression, which suggests that suspiciousness alone is as good as assessing all eight non-suspicious schizotypy features together. This study also provides evidence suggesting that adolescent’s with high levels of non-suspicious schizotypy and suspiciousness suffer from high levels of peer problems which in turn can result in low self-esteem and lead them to be reactively aggressive rather than proactively aggressive toward others, a theoretical model invariant across gender and age that deserves to be tested further with a longitudinal design. This study’s finding has implications on future research, suggesting that interventions aimed at enhancing self-esteem in adolescent’s with high schizotypal traits during middle childhood may in turn reduce retaliatory reactive aggressive behaviors toward others.
